# New Insights into the Role of Cysteine Cathepsins in Neuroinflammation

**DOI:** 10.3390/biom11121796

**Published:** 2021-11-30

**Authors:** Anja Pišlar, Lara Bolčina, Janko Kos

**Affiliations:** 1 Faculty of Pharmacy, University of Ljubljana, Askerceva cesta 7, 1000 Ljubljana, Slovenia; lara.bolcina@ffa.uni-lj.si (L.B.); janko.kos@ffa.uni-lj.si (J.K.); 2 Department of Biotechnology, Jožef Stefan Institute, Jamova cesta 39, 1000 Ljubljana, Slovenia

**Keywords:** microglia, cysteine cathepsins, neurotoxic polarization, neuroinflammation, neurodegeneration

## Abstract

Neuroinflammation, which is mediated by microglia and astrocytes, is associated with the progression of neurodegenerative diseases. Increasing evidence shows that activated microglia induce the expression and secretion of various lysosomal cathepsins, particularly during the early stage of neuroinflammation. This trigger signaling cascade that aggravate neurodegeneration. To date, most research on neuroinflammation has focused on the role of cysteine cathepsins, the largest cathepsin family. Cysteine cathepsins are primarily responsible for protein degradation in lysosomes; however, they also play a role in regulating a number of other important physiological and pathological processes. This review focuses on the functional roles of cysteine cathepsins in the central nervous system during neuroinflammation, with an emphasis on their roles in the polarization of microglia and neuroinflammation signaling, which in turn causes neuronal death and thus neurodegeneration.

## 1. Introduction

Neuroinflammation is a common mechanism that plays a crucial role in the pathogenesis of various neurodegenerative diseases [1,2]. Primarily, it is a defense mechanism that protects the brain from diverse pathogens [3]. However, it may trigger both beneficial and detrimental effects on neurons [2,4,5]. Much evidence points to the neuroinflammatory effects of glia cells, supporting cells for neurons in the central nervous system (CNS) [6,7]. Studies have indicated that neuroinflammation is actually beneficial in certain circumstances, secreting neurotrophic factors that prevent neural injury, stimulate myelin repair, and remove toxic aggregated proteins and cell debris from the CNS [8,9,10,11]. Persistent inflammatory responses, however, are detrimental and involve glia activation, subsequently leading to neurodegenerative diseases such as Parkinson’s disease, Alzheimer’s disease (AD), and amyotrophic lateral sclerosis [2,12,13,14].

**Figure 1:**
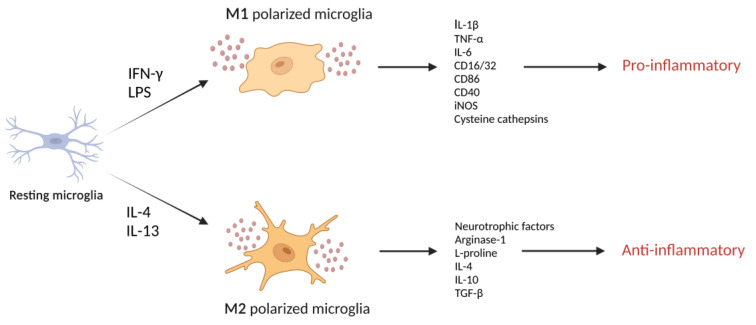
Scheme of M1-like or M2-like polarization of microglia. Microglia polarize the M1-like state (classic, proinflammatory) following stimulation of lipopolysaccharide (LPS) or interferon (IFN)-γ, whereas microglia polarize M2-like state (alternative, anti-inflammatory, protective) by protective cytokines such as interleukin (IL)-4 or IL-13. The M1-like responses are shown by the upregulation of pro-inflammatory cytokines such as interleukin IL-1β, IL-6, tumor necrosis factor (TNF)-α, cluster of differentiation (CD)16/32, CD86, CD40, inducible nitric oxide synthase (iNOS), and cysteine cathepsins. The M2-like responses are shown by the upregulation of markers such as arginase-1, L-proline and anti-inflammatory cytokines, IL-4, IL-10 and transforming growth factor (TGF)-β, and neurotrophic factors.

Glia cells, consisting of microglia, astrocytes, and oligodendrocyte lineage cells, are superior to neurons in cellular diversity and function and are considered neuron-supporting cells [15]. In the CNS, microglia represent resident innate immune cells, and their contribution to inflammatory signaling pathways between neurons, astrocytes, and other components of brain tissue largely sets the magnitude of the immune response [16,17]. Microglia in the brain are highly plastic and can adopt distinct phenotypes (Figure 1), including the classically (M1) and alternatively (M2) activated states in response to various stimuli [18]. During inflammation, polarized M2 microglia secrete anti-inflammatory mediators and neurotrophic factors that are involved in restoring homeostasis, whereas polarized M1 microglia produce pro-inflammatory cytokines, neurotoxic molecules that contribute to neural network dysfunction and promote inflammatory reactions [19]. Characterizing and understanding the mechanism of action of endogenous biomolecules involved in detrimental neuroinflammation may be critical for the development of novel therapeutic strategies for treating neurodegenerative diseases that involve neuroinflammation.

Peptidases have been identified as important players in chronic innate neuroinflammation mediated by microglia and astrocytes ultimately associated with the progressive nature of neurodegenerative disorders [20,21]. Peptidases catalyze the hydrolysis of peptide bonds, linking amino acid residues in the polypeptide chain. They belong to seven main groups according to the characteristics of their catalytic site: serine, cysteine, threonine, aspartyl, glutamic, asparagine and matrix metallopeptidases [22]. Proteolysis is stringently regulated in biological systems by various mechanisms, including: regulation of their expression at transcriptional and translational levels; specificity of the catalytic cleft; synthesis of peptidases as inactive zymogens; their compartmentalization away from their substrates and their release only after a specific signal; activation by co-factors; and, finally, by irreversible or reversible suppression of proteolytic activity by protease inhibitors which prevents access of the substrate to the catalytic cleft [23]. Any dysregulation in the peptidase expression and/or their proteolytic activity disrupts cellular homeostasis, leading to pathological conditions [24]. Namely, matrix metallopeptidases receive great attention as mediators of neuroinflammation. They are widely distributed in the brain and regulate various processes including microglial activation and tissue degradation through processing of pro-inflammatory cytokines [21,24,25]. On the other hand, certain aspartic and cysteine peptidases have been shown to play important roles in the major histocompatibility complex class II-mediated antigen presentation of microglia, or in clearance of phagocytosed amyloid-beta peptides [21]. Additionally, increasing evidence shows that activated microglia express and secrete lysosomal cathepsins, particularly during the early stage of neuroinflammation, which triggers signaling cascades that aggravate neurodegeneration [26,27,28,29]. Therefore, this review highlights new insights into the roles of lysosomal peptidases, particularly cysteine cathepsins during neuroinflammation.

## 2. Lysosomal Peptidases

Lysosomal peptidases represent lysosomal hydrolytic enzymes that catalyze the breakdown of proteins [30,31]. They are predominantly located within endosomes/lysosomes but can also be found in the nucleus, cytosol, cell membrane, or extracellularly [31,32]. According to the site of peptide bond hydrolysis, they can be divided into endo- and exo-peptidases. Most of them preferably function as endopeptidases, by cleaving peptide bonds within a polypeptide chain, whereas only a few peptidases function as exopeptidases. The latter can cleave polypeptide protein chains at the N-terminal (aminopeptidases) or C-terminal end (carboxypeptidases) [30,33]. Depending on the catalytic group in the active site, they belong to the aspartic, serine, or cysteine peptidase families of hydrolytic enzymes [22].

The name cathepsin derives from the Greek verb katahepsein (“to digest”) and was first proposed for peptidases active in slightly acidic environments but was later used for all aspartic, serine, and cysteine cathepsins [31,34]. Because of their proteolytic activity, cathepsins are integral in many biological processes, such as the immune response, cell cycle, apoptosis, antigen presentation, phagocytosis, prohormone activation, and metabolism [31,33,35]. Peptidase expression and activity are regulated to avoid any disruption of cellular homeostasis. Since peptidases catalyze the irreversible cleavage of peptide bonds, any dysregulation in their expression, localization, or proteolytic activity can result in pathological processes. Therefore, peptidase signaling pathways are strictly controlled by several mechanisms: through the regulation of gene expression; post-translational modifications; zymogen activation; accessibility of the susceptible peptide bond in the substrate; and, ultimately, their endogenous inhibitors [36].

### 2.1. Cysteine Peptidases

**Figure 2:**
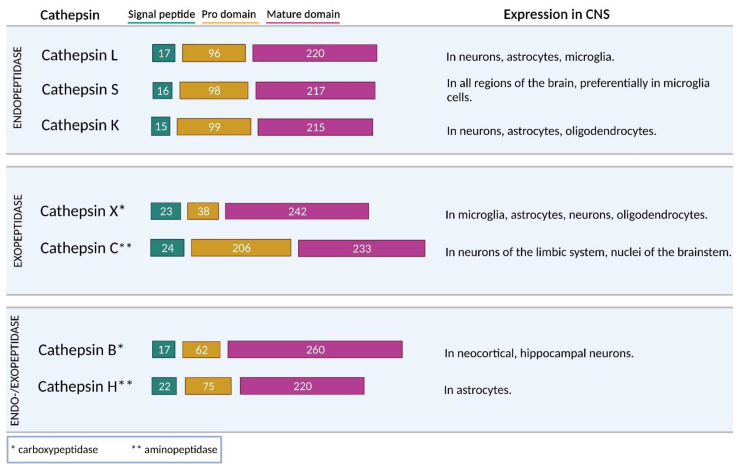
Schematic representation of human cysteine cathepsins structure with significant expression in the CNS. Representation of cysteine cathepsins functional sequence according to their number of amino acids, length of domains (signal peptide, prodomain, and mature domain) together with peptidase expression patterns in cells of the CNS. The signal peptide is cleaved off at the site of the translocation into the endoplasmic reticulum. The propeptide is cleaved in the increasingly acidic environment of the endosomal/lysosomal system where it is converted to the active form under acidic conditions, autocatalytically, or by other peptidases. According to the site of peptide bond hydrolysis, cathepsins can act as endo- and/or exo-peptidases.

The most extensive group of peptidases are cysteine peptidases, which includes 11 cathepsins (cathepsins B, C, F, H, K, L, O, S, V, X, and W) [22]. They belong to the cysteine peptidase clan CA and are widely distributed among living organisms [22,31]. Most cysteine cathepsins (F, K, L, O, S, and V) are predominantly endopeptidases, whereas cathepsins C and X are exclusively exopeptidases (Figure 2). Cathepsin C has aminopeptidase activity, and cathepsin X has carboxypeptidase activity. Furthermore, cathepsins B and H exhibit both endo- and exo-peptidase activity. Apart from their endopeptidase activity, cathepsins B and H also act as carboxypeptidases and aminopeptidases, respectively [37].

The expression of cysteine cathepsins depends on tissue distribution and their biological function. Most cathepsins are ubiquitously expressed in human tissues, while others have more specific distributions. For example, cathepsin K is mainly expressed in osteoclasts, osteoblasts, and epithelial cells and is involved in bone resorption [38]. Cathepsin S is restricted to antigen-presenting cells derived from bone marrow and plays a role in the major histocompatibility complex class II antigen-presenting pathway [39]. Cathepsin W is expressed exclusively in CD8 T-lymphocytes and natural killer cells [40]. Cathepsin V is found in the thymus, testis, and cornea [41]. Cathepsin X is expressed in immune cells such as macrophages, dendritic cells, microglia, B lymphocytes, and natural killer cells [42,43].

Cathepsins are synthesized as inactive preproenzymes. Cleavage of the signaling protein and N-glycosylation of the remaining pro-cathepsin occurs during the passage to the endoplasmic reticulum. Glycosylated pro-cathepsin then enters lysosomes via the mannose-6-phosphate receptor pathway, where it is converted to the active form by the acidic conditions, autocatalytically, or by other peptidases [44]. Optimal cathepsin function and stability occurs at a slightly acidic pH within lysosomes, whereas cathepsin function is lost at neutral pH in the cytosol and extracellular environment. An exception is cathepsin S [45], which is stable at a neutral or slightly alkaline pH [31]. Furthermore, the proteolytic activity of cathepsins can be increased or decreased due to pathological abnormalities. Reduced activity most often originates from genetic defects, excessive inhibitory activity, or limited activation. By contrast, enhanced proteolysis is, in addition to genetic causes, a consequence of the endogenous and/or exogenous action of various factors that trigger signaling pathways and result in unwanted peptidase activation. Increased proteolytic activity has been detected in bacterial, viral, parasitic, cardiovascular, inflammatory, and neurodegenerative diseases as well as in cancer [33]. Increasing evidence suggests that disturbance of the normal balance of enzymatic activities is the first insult in brain aging and age-related diseases [21].

### 2.2. Cysteine Cathepsins in the CNS

Although all cathepsins are targeted to endosomes/lysosomes, they are not equally distributed among different tissues [46,47]. Variations in cathepsin concentration and distribution in the CNS have been reported in normal aged brains [48]. Some cathepsins, e.g., cathepsins B, H, L, C, and O, are ubiquitously present in various tissues and cells (including the brain), whereas other cathepsins, e.g., cathepsins F, K, S, V, X, and W, show more limited cell and tissue distribution and expression. However, all of them, except cathepsin W, were found to be involved in CNS biology and pathology [46,47]. Increasing evidence indicates that cathepsins, including cathepsins B, L, H, C, S, and X, play important roles in CNS diseases. During neurodegeneration, cathepsins contribute to neuronal injury induced by excitotoxins, through degradation of axonal and myelin proteins, by converting protein precursor into active peptide neurotransmitters and by amplifying apoptotic signaling [49,50]. Furthermore, a central role in the neuronal cell death mechanism has been proposed for cathepsins [37,51]. Cysteine cathepsins were identified as hallmarks of aging and neurodegeneration with a role in oxidative stress, mitochondrial dysfunction, abnormal intercellular communication and dysregulated trafficking, and deposition of protein aggregates in neuronal cells. However, again, their deficiency may result in other pathological states such as lysosomal storage disease [47]. Therefore, their role is CNS is discussed in more detail.

Of all the cathepsins, **cathepsin B** is the most studied cathepsin expressed in the CNS. It is found in different brain regions, preferentially in neocortical and hippocampal neurons [52,53]. Its proteolytic activity seems to be important in neuronal development [54] and cell proliferation [55]. It engages in digesting proteins, chromatin, lipids, and carbohydrates and physiological protein turnover in neurons [21,48]. Increased cathepsin B levels were found to be responsible for the activation of nuclear factor-κB (NF-κB) [56] and the degradation of mitochondrial transcription factor A in aged microglia [57]. Cathepsin B is also involved in inducing apoptosis by activating pro-caspases-1 [58] and -11 [59] and cleaving the Bcl-2 family member Bid [60]. It plays an important role in the proteolytic cascade of the breakdown of connective tissue within the extracellular matrix (ECM) [61,62,63] and in the shedding of integrins [64]. In chromaffin cells, cathepsin B is associated with the production of neurotoxic amyloid β (Aβ) peptides [65,66,67].

**Cathepsin L** is abundantly distributed throughout different brain areas and is preferentially expressed in neurons [68], astrocytes, and microglia [69]. In the CNS, it mediates cell–cell communication as it participates in the biosynthesis of peptide neurotransmitters [70]. It cleaves pro-neuropeptide Y and is consequently involved in the production of mature pro-neuropeptide Y in cortical neurons and neuronal chromaffin cells [68]. It is also involved in the production of other neurotransmitters, including enkephalin, dynorphin, and cholecystokinin [70]. Extracellular cathepsin L plays an important role in tissue remodeling in vitro, as it stimulates axonal growth in cortical and spinal cord neurons [71] and is also involved in cleaving ECM proteins [62].

A novel role for **cathepsin H** in pro-neuropeptide processing has been demonstrated. Cathepsin H has been associated with neuropeptide-producing secretory vesicles due to its cleavage of N-terminal basic residues of (Met)enkephalin [72]. Conversely, cathepsin H can also act as an endopeptidase, metabolizing neuropeptides and bradykinin [73]. In human tissues, it is mainly found in secretions and extracellular fluids, with high concentrations in cerebral spinal fluid [74]. It is distributed in all regions of the brain and is mainly produced by astrocytes [75]. Apart from its involvement in the metabolism, production, and inactivation of neuropeptides [73], a recent study demonstrated its involvement in Toll-like receptor 3 (TLR3)/interferon β (IFN-β) signaling. Cathepsin H deficiency decreases hypoxia–ischemia-induced hippocampal atrophy in neonatal mice by affecting TLR3/IFN-β signaling [76].

**Cathepsin C** is expressed in a variety of mammalian tissues, with the highest levels in lungs, kidneys, liver, and spleen, but relatively low levels in the brain. The distribution of cathepsin C in normal brain is restricted to the neurons of the limbic system and several nuclei of the brainstem. Its granular immunohistochemical signals were found in neuronal perikarya of particular brain regions, such as the accessory olfactory bulb, septum, CA2 of the hippocampus, part of the cerebral cortex, medial geniculate, and inferior colliculus [77,78]. Cathepsin C is predominantly induced in activated microglia following systemic injection of lipopolysaccharide (LPS) or stimulation with factors such as interleukin 1β (IL-1β) and interleukin 6 (IL-6) [78]. Cathepsin C can regulate normal neuronal functions, e.g., by inducing glia-derived chemokine ligand 2 production, which attracts inflammatory cells to sites of myelin sheath damage in a cuprizone model [79].

**Cathepsin S** is unique within the cysteine cathepsin family due to its ability to retain activity at a neutral pH, which increases its potential involvement in extracellular proteolytic processes [80,81]. It is expressed in all regions of the brain, preferentially localizes in microglia cells, and is an important player in microglia-neuron communication [82]. Cathepsin S is released by microglia and macrophages following stimulation with inflammatory cytokines and pro-inflammatory LPS [21,48,82,83]. Because of its extracellular activity, it can degrade several ECM components and support microglial migration to inflammation sites in the CNS [84,85]. For instance, it cleaves several ECM molecules, including fibronectin, laminin, neurocan, and phosphacan [86]. Cathepsin S-induced microglial migration also protects facial motoneurons against axotomy-induced injury [87]. Furthermore, cathepsin S activity is critical for invariant chain degradation in antigen-presenting cells [88], including dendritic cells and microglia, and therefore plays an important role in antigen presentation [87].

Another cathepsin that has been studied for its role in inflammation-associated neurodegenerative diseases is **cathepsin X**. Cathepsin X is an important cysteine peptidase in degenerative processes during normal aging and in neurodegenerative diseases. It is primarily expressed in cells of the immune system, such as monocytes, macrophages, and dendritic cells, but also widely expressed in brain cells, which implies its involvement in neuroinflammatory processes. In the brain, it is localized in microglia, astrocytes, aged neurons, and even oligodendrocytes [43,46,89]. With its proteolytic activity in neuronal cells, cathepsin X cleaves C-terminal amino acids of γ-enolase and consequently abolishes its neurotrophic activity [42,90]. Additionally, cathepsin X inhibition increases plasmin generation, which is essential for neuronal differentiation and changes the length distribution of neurites, especially in the early phase of neurite outgrowth. Moreover, cathepsin X inhibition increases neuronal survival and reduces apoptosis induced by serum deprivation, particularly in the absence of nerve growth factor [90].

Apart from all of the mentioned roles of cathepsins in CNS, **cathepsin K** expression was also observed in multiple neurons as well as in astrocytes and white matter oligodendrocytes [91]. Several studies have described the presence of cathepsin K in the CNS of rats and post-mortem human brain tissue [91,92]. It is capable of cleaving neuroactive peptides (i.e., bradykinin and other kinins) and plays a role in processing β-endorphin to release met-enkephalin [91]. Furthermore, it was suggested that cathepsin K deficiency in mice has a multiple-level impact on brain development and metabolism. In the brain of cathepsin K-deficient animals, the analysis of neuronal markers demonstrated that the architecture of the neuronal layers was affected by cathepsin K deficiency in the hippocampus, a region important in regulation of anxiety and memory. The study also confirmed a clear impact of cathepsin K deficiency on learning and memory [93]. Cathepsin K activity is enhanced also in schizophrenia [91].

## 3. Processes Triggered by Microglial Cathepsins in the Brain

Inflammatory processes in the CNS are directly involved in neuronal death and therefore in the development of neurodegenerative diseases such as Alzheimer’s and Parkinson’s disease. Microglia are the resident innate immune cells in the brain and actively contribute to neuronal damage in neurodegenerative diseases [6]. Various stimuli can activate microglia, including endogenous pro-inflammatory mediators, such as tumor necrosis factor α (TNF-α), IL-1β, interferon γ (IFN-γ), Aβ peptide, and exogenous pathogenic bacteria or viruses [84,94]. Chronic microglia activation causes neuronal damage by excessive release of potentially toxic molecules, e.g., pro-inflammatory cytokines (TNF-α and IL-1β), nitric oxide, and reactive oxygen species [95,96], and by inducing the synthesis and secretion of lysosomal cathepsins [21,84]. Pro-inflammatory mediators released by activated microglia cells rapidly trigger further activation of surrounding microglia cells and astrocytes [84]. Additionally, dying neurons release factors that maintain further microglia activation. This creates a cyclical relationship between microglia activation and neuronal death, contributing to neurodegeneration [84].

### 3.1. Cysteine Cathepsins Convert Protein Precursors into Toxic Peptides

One of the major proteins responsible for the formation of amyloid plaques and toxic fragments in AD is neurotoxic Aβ, a polypeptide formed by the proteolytic cleavage of amyloid precursor protein (APP) [65] in secretory vesicles of neuronal chromaffin cells [97]. Furthermore, β- and γ-secretases are largely responsible for the proteolytic cleavage of APP into Aβ, and cathepsins B, L, and S exhibit β-secretase activity [98]. Among them, cathepsin B is the most common β-secretase for the production of neurotoxic Aβ peptide [67,99,100]. Immunoelectron microscopy revealed colocalization of cathepsin B and Aβ in secretory vesicles [97]. Furthermore, selective cathepsin B inhibitor CA074 and its cell-permeant analog CA074Me prevent Aβ production from endogenous APP in isolated secretory vesicles, resulting in reduced Aβ release from neuronal cells. CA074Me also reduces the production of APP-derived COOH-terminal-β-secretase-like cleavage product, suggesting that cathepsin B acts as a β-secretase in secretory vesicles of neuronal chromaffin cells [97]. Another study, using a transgenic mouse model containing the wild-type β-secretase site sequence of APP that is present in most AD patients, showed that cathepsin B gene deletion improves memory deficits and reduces brain amyloid plaque load [66]. Deletion of the cathepsin B gene also reduces amyloid plaque load and decreases Aß_1-40_ and Aß_1-42_ [66,101]. Aβ_1-40_ and Aβ_1-42_ are two of several forms of Aβ that have the same N-terminal but differ in their C-terminal residues [101]. Cathepsin B preferably cleaves wild-type β-secretase substrate, but not Swedish mutant substrate, which explains why E-64d-induced cathepsin B inhibition had no effect in AD mice expressing the Swedish mutant β-secretase site of APP [100].

In addition to the pathogenic role of cathepsin B in aging and age-related neurodegeneration, cathepsin B seems to degrade Aβ via C-terminal truncation, thus leaving its involvement in Aβ metabolism unclear [102,103]. It has been demonstrated that cathepsin B actually reduces Aβ peptide levels, especially the aggregation-prone species Aβ_1-42_ [103]. Furthermore, a study on cultured astrocytes showed different effects of cathepsin B on Aβ regulation that might depend on the cellular localization of active cathepsin B. Non-lysosomal cathepsin B mediates Aβ production in astrocytes, while Aβ degradation seems to depend on lysosomal cathepsin B and the production of Aβ peptides [104]. This emphasizes the need to consider organelle targeting in drug development that promotes Aβ degradation and clearance [104].

Besides cathepsin B, also cathepsins L and S have been identified to cleave the wild-type β-secretase site [98]. Cathepsin L levels in the brain are similar to that of cathepsin B. High levels of cathepsins L were found in neurons and amyloid plaques in the brain of AD patients [105]. It has been demonstrated that human cathepsin L cleaves the human wild type β-secretase site sequence 74-fold better than β-site APP cleaving enzyme 1. On the other hand, human cathepsin S cleaves the human wild type β-secretase site sequence 1170-fold better than β-site APP cleaving enzyme 1 [98]. Cathepsin S expression in a normal brain is very low but is induced in the brains of AD patients. Cathepsin S may be relevant to the pathogenesis of AD, since transfecting human kidney cells with cathepsin S increases the secretion of modified Aβ into the culture medium. Furthermore, Aβ secretion was blocked with the cysteine peptidase inhibitor E-64d [106]. Cathepsin S also takes part in the clearance of Aβ peptides in vivo, both intracellularly or extracellularly, since it is able to degrade monomeric and dimeric Aβ peptides at both acidic and neutral pH [83]. Therefore, it modulates peptide levels at the very initial stages of peptide aggregation, which in turn might have an effect on Aβ neurotoxicity [107].

### 3.2. Cysteine Cathepsins Play an Important Role in Neural Tissue Remodeling

The localization of cathepsins is not restricted to only intracellular compartments but can also be found in the extracellular space, indicating their broad spectrum of biological activities [63,108,109]. Several mechanisms participate in lysosomal permeabilization, which results in the release of lysosomal enzymes into the cytosol. The amount of potentially hazardous proteolytic enzymes, mainly cathepsins, in the cytosol may overcome the protective inhibitory effect of endogenous protein inhibitors [44,108]. The extracellular localization of cysteine cathepsins is often associated with their increased expression that results in pathological conditions [21,48,108]. Cysteine cathepsins are the major cysteine proteases involved in ECM reorganization and the nonspecific degradation of ECM proteins [44,110].

Upon stimulation with inflammatory mediators, cathepsin S can be released from activated microglia [21,48,86]. Among all the cathepsins, cathepsin S is the most suited for such extracellular processes, as it retains most of its proteolytic activity at neutral pH [86]. This feature enables cathepsin S to degrade proteins outside of the lysosomal compartment, including ECM components, and to support microglial migration in the CNS [86]. Cathepsin S cleaves many components of the ECM, e.g., fibronectin, laminin, neurocan, fosfacan, and heparan sulfate proteoglycans [86]. In AD brains, heparan sulfate proteoglycans are components of the senile plaques that can protect the potentially neurotoxic Aβ peptide from proteolysis. Moreover, the proteoglycan protein moiety is critical for amyloid fibril formation and persistence. It is therefore possible that cathepsin S plays a modulatory role in the formation and persistence of amyloid fibrils in senile plaques [86]. Furthermore, cathepsins B and L play a role in cleaving the heparan sulfate proteoglycan perlecan. The latter is a key component of the ECM and is involved in generating a C-terminal LG3 fragment with neuroprotective roles. Thus, perlecan could represent one of the defense mechanisms against ischemic injury [110].

Cathepsins also contribute to damaging neurons and oligodendrocytes by their proteolytic action on axons and myelin proteins [50,111]. It was demonstrated that the balance between cathepsins and their inhibitor cystatin C changes during the course of demyelination, possibly leading to cytotoxic effects on neurons (axons) and oligodendrocytes [111]. Conversely, extracellular cathepsin L has an important role in tissue remodeling in vitro, as it stimulates axonal growth in cortical and spinal cord neurons [71].

### 3.3. Cysteine Cathepsins Induce Neuronal Death

Neuronal death is normal during nervous system development but can become devastating if not regulated, e.g., neuronal degeneration in chronic neurodegenerative diseases. It can be divided into necrosis and apoptosis and is regulated by several proteins and signal-transduction pathways. Players in the cell death and cell survival orchestra include: Fas receptor; Bcl-2 and Bax cytochrome c; caspases; p53; and extracellular signal-regulated protein kinases [112]. Lysosomes and lysosomal peptidases, including cathepsins, have often been linked with cell death. It is now clear that cysteine peptidases from the caspase family play a major role in neuronal apoptosis and that their activation is a critical step in apoptosis [113].

As already discussed, cathepsins are either localized intracellularly (within lysosomes) or extracellularly [48] and are differentially expressed in microglia in response to pro-inflammatory stimuli [83,94]. Factors released by microglia can kill neurons directly by promoting neuronal self-destruction or indirectly by promoting non-neuronal cells to produce other factors that induce neuronal death [94]. Lysosomal peptidases have been suggested to interfere with the apoptotic cascade in microglia by cleaving and activating caspases [114]. Secreted cathepsin B is a major causative factor of microglia-induced neuronal apoptosis. It cleaves pro-caspases-1 and -11, which are, however, only indirectly implicated in the apoptotic process [114]. Cathepsin B affects the production of the mature cytokine IL-1β by proteolytically activating pro-caspase-1. IL-1β is then rapidly secreted from microglia by exocytosis [58] and involved in the release of reactive nitrogen and oxygen species from microglia and in the mediation of microglial activation and proliferation. Therefore, IL-1β production may enhance microglial inflammatory responses and cause neuronal apoptosis [115]. Furthermore, cathepsin B is also involved in activating pro-caspase-11. Active caspase-11 is important for pro-caspase-1 activation and thus pro-IL-1β maturation [59].

**Figure 3:**
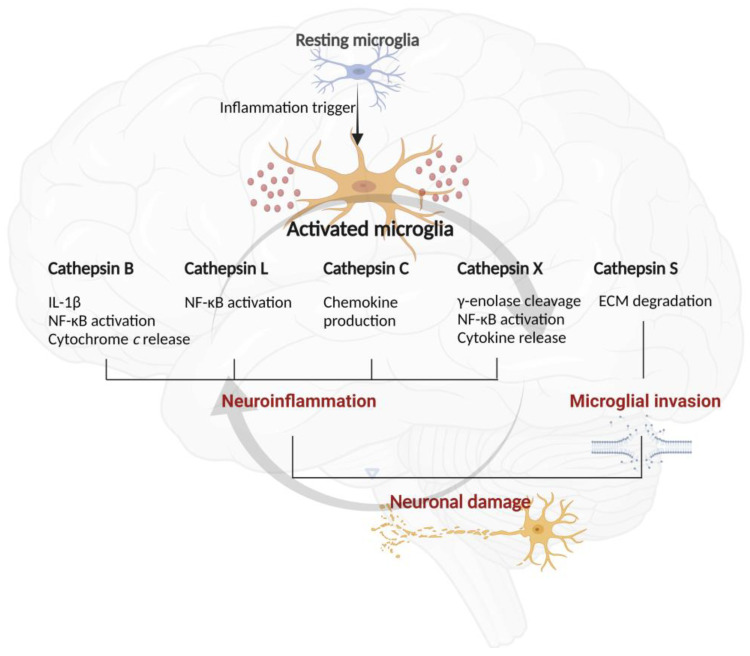
The involvement of microglial cathepsins in inflammation-induced neurodegeneration. Cysteine cathepsins can be released from activated microglia following various toxic insults, and their activity through various pathways may be lethal to neurons. Extracellular cathepsins B, L, and X promote inflammation through the nuclear factor-kappa B (NF-κB) pathway. Cathepsin B also promotes inflammation by participating in the maturation of interleukin-1β (IL-1β) and cleavage of pro-apoptotic Bid protein, which leads to the release of cytochrome *c*. Cathepsin X also cleaves the C-terminal end of γ-enolase, abolishing its neurotrophic activity, and releases pro-inflammatory cytokines. Cathepsin C induces chemokine production. Extracellular cathepsin S assists microglial migration by degrading extracellular matrix (ECM) components. The relationship between microglia activation and neuronal death is cyclical.

Cathepsins are also involved in caspase activation and thus neuronal degradation by cleaving Bid protein. The cysteine peptidases B, H, K, L, and S are involved in cleaving pro-apoptotic Bid protein, a member of the Bcl-2 family, leading to the mitochondrial release of cytochrome *c* that causes apoptotic caspase activation [47,116]. Bid cleavage results in the formation of truncated (t)BID that, after lysosomal leakage, activates Bax and Bak proteins by promoting their oligomerization to form pores in the outer mitochondrial membrane. Cytochrome *c* exits mitochondria through these pores and, once in the cytosol, activates caspase-9 and thus promotes the executioner caspases-3 and -7 [117]. The main roles of these cathepsins in microglial functions are illustrated in Figure 3.

Cathepsin H is involved in lysosomal protein degradation [118,119]. The neuropathological role of cathepsin H was investigated in an LPS-induced neuroinflammation cell model [120]. It was demonstrated that the percentages of apoptosis and necrosis in cathepsin H-treated cells were significantly higher than those of control cells. This indicates that cathepsin H could have a neurotoxic influence on neurons resulting in neuronal death; however, the exact mechanism remains to be determined. It is suggested that secreted cathepsin H may function as a ligand, directly binding to yet unidentified specific receptors on the surfaces of neurons, triggering intracellular death-related signaling pathways [120].

Additionally, cathepsin X promotes the apoptosis of neuron-*like* cells induced by 6-hydroxydopamine by activating the caspase cascade [121]. Substantially increased cathepsin X secretion from microglia has been observed in response to inflammatory stimuli, leading to microglia activation-mediated apoptosis and cell death of neuron-*like* cells [42]. Overall, these findings indicate that cysteine cathepsins play an important role in neuroinflammation, as microglia activation and excessive cathepsin release lead to neuronal cell death.

## 4. Cysteine Cathepsin as a Key Player in Neuroinflammation

Accumulating evidence suggests that neuroinflammation mediated by microglia and astrocytes is involved in the progressive nature of neurodegenerative disorders [13]. Neuroinflammation can be divided into acute and chronic phases [122]. Inflammatory stimuli activate glia cells, which release inflammatory cytokines and phagocytose debris and dead cells to initiate tissue repair and, thus, resolve inflammation. However, the persistence of the initiating factors, such as injury, infection, exposure to a toxin, or a failure of mechanisms required for resolving the inflammatory response, result in a self-propagating and persistent stage of chronic inflammation. The latter leads to neuronal toxicity, accompanied by oxidative stress [123,124], mitochondrial dysfunction [125], and activation of the apoptotic cascade [126,127]. This finally leads to aggressive neuronal loss and neurodegeneration [128,129,130,131]. In addition to inflammatory molecules, activated microglia also secrete lysosomal peptidases, which support various microglial immune functions and key inflammatory pathways [21,84,132]. These lysosomal peptidases also include cysteine cathepsins [27,29,83,86,133].

### 4.1. Microglial Cathepsins in Neuroinflammation-Induced Neurodegeneration

Overactivation of microglia and excessive amounts of released pro-inflammatory cytokines by microglia might result in neurotoxic consequences in neurodegenerative diseases. Increasing evidence indicates that microglial activation is an early and ongoing event in several neurodegenerative diseases [21]. Several studies using in vivo models of neurodegeneration demonstrated marked increases in the expressions of cathepsins B [134], L [135], H [120], C [78], and X [136] in different brain regions following LPS-induced neuroinflammation. Among them, microglial cathepsin B has been extensively studied. Cytoplasmic cathepsin B acts as a pro-inflammatory factor as it enhances the activation of caspase-1 and consequently the production and secretion of IL-1β [58] through pyrin domain-containing protein 3 inflammasome-independent processing of pro-caspase-3 in phagolysosomes [137]. Cathepsin B leakage from the endosomal/lysosomal system during aging is associated with the proteolytic degradation of mitochondrial transcription factor A, which can stabilize mitochondrial DNA. Therefore, microglial cathepsin B may function as a major driver of inflammatory brain diseases and brain aging (reviewed in [132]). Indeed, secreted cathepsin B has been shown to be a major causative factor of microglia-induced neuronal apoptosis [27]. During LPS-induced inflammation, cathepsin B is also translocated from lysosomes to other subcellular compartments in hippocampal neurons [138].

Similarly, the expression of microglia-secreted cathepsin C is enhanced during CNS inflammation. Cathepsin C expression in the brain is predominantly induced in activated microglia [78], and microglial cathepsin C plays a role in promoting chemokine production during brain inflammation [79]. Similarly, the expression of microglia-secreted cathepsin S is also enhanced during CNS inflammation and aging in mice [82].

Altered cathepsin S expression is controlled by a built-in molecular clock in cortical microglia, and the circadian expression of cathepsin S is involved in diurnal variations of neuronal synaptic strength via proteolytic modification. Cathepsin S has also been associated with some sleeping disorders, as its genetic ablation causes reduced synaptic strength during sleep by inducing hyperlocomotor activity, which is required to obtain novel information after waking [139].

Cathepsin L is also reported to play important roles in neuroinflammation-induced neurodegeneration. Cathepsin L is widely distributed throughout the CNS and is involved in the activation of microglia, an important source of cathepsin L [140]. Cathepsin L is upregulated in the *substantia nigra pars compacta* (SNc) of patients with Parkinson’s disease [141]. Substantially increased cathepsin L secretion from microglia has also been observed in response to LPS, supporting its role in contributing to inflammatory responses [142].

Cathepsin H importantly contributes to peripheral inflammatory pathologies [120]. It has been implied that cathepsin H provokes acute inflammation characterized by the accumulation of polymorphonuclear leukocytes when it is injected intracutaneously into newborn rats [143]. Cathepsin H immunoreactivity in the hippocampus is increased in an animal model of cerebral ischemia, and cathepsin H activity increases in affected brain areas in Huntington’s disease [118]. A recent in vivo study showed a prominent upregulation of cathepsin H expression in brain microglia after LPS injection, supported by an in vitro study confirming a potential role of cathepsin H in the neuroinflammatory pathogenesis of neurological diseases [120].

**Figure 4:**
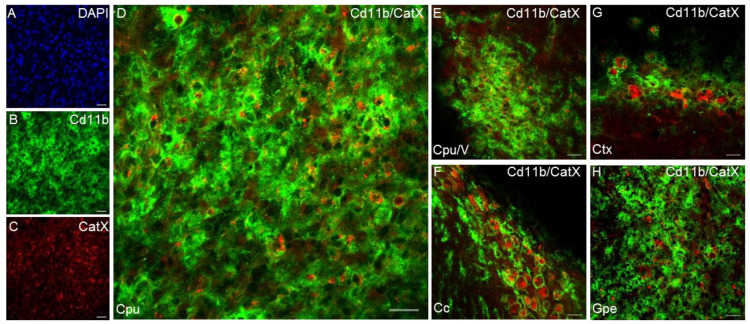
Microglial upregulation of cathepsin X in rat brain after intrastriatal LPS injection. Representative images of double immunofluorescence staining of microglial marker Cd11b (green fluorescence; **B**) and cathepsin X (red fluorescence; **C**) in the ipsilateral side of the striatal brain slices 4 weeks after LPS-induced lesion. Nuclei were counterstained with DAPI (blue fluorescence; **A**). In the striatum (Cpu; **D**), caudate-putamen/ventricular (Cpu/V; **E**), corpus callosum (Cc; **F**), cortex (Ctx; **G**), and external globus pallidus (GPe; **H**), upregulated cathepsin X was predominantly restricted to Cd11b-positive cells. However, some cathepsin X-positive signal did not overlap with Cd11b-positive signal in Cpu/V and GPe. Images were acquired using an LSM 710 Carl Zeiss confocal microscope and ZEN imaging software. Scale bars, 20 µm.

Another cysteine cathepsin with an inflammatory role in the CNS is cathepsin X [89]. The expression and proteolytic activity of cathepsin X were strongly upregulated in the degenerating brain regions in a transgenic mouse, especially in glial cells and aged neurons [89,144]. Cathepsin X is disproportionately expressed and secreted by activated microglia and astrocytes in response to neuronal damage and inflammatory stimuli, both in vitro and in vivo [29,42,145,146]. In vitro, substantially increased cathepsin X secretion from microglia has been observed in response to the inflammatory stimulus induced by LPS, leading to microglia activation-mediated neurodegeneration [29,42]. In vivo, unilateral LPS injection into the striatum increased cathepsin X expression and activity in the striatum and surrounding areas on the ipsilateral side. In addition to the striatum, cathepsin X overexpression was detected in other brain areas such as the cerebral cortex, corpus callosum, subventricular zone, and external globus pallidus, and prominent upregulation was mainly restricted to activated microglia and reactive astrocytes (Figure 4). Moreover, the administration of a cathepsin X inhibitor along with LPS injection revealed its potential protective role in neuroinflammation-induced striatal lesions [136]. Additionally, dendritic cells in aging mouse brains had increased cathepsin X protein levels, which correlated with known markers of neuroinflammation [89]. Allan et al. showed that cathepsin X-deficient mice have reduced neuroinflammation and circulating IL-1β levels during experimental autoimmune encephalomyelitis [147].

### 4.2. Cathepsins Promote Neurotoxic Polarization of Microglia

During inflammation, M1-polarized microglia, evoked by exposure to IFN-γ or bacterial toxins (e.g., LPS), are characterized by an ameboid shape, high mobility, strong phagocytic activity, the production of pro-inflammatory mediators (e.g., IL-1β, TNF-α, and IL-6), and an increased expression of surface markers (e.g., CD16/32, CD86, CD40, and inducible nitric oxide synthase), which fuel the inflammatory process [148]. In vitro exposure to inflammatory stimuli (e.g., LPS) increases the levels of certain cysteine cathepsins in culture supernatants of the microglia cell line BV2 [29,142]. Substantially increased cathepsin L secretion from microglia has been observed in response to LPS treatment for 1 h, which is earlier than the upregulation of pro-inflammatory cytokines. This indicates that the earlier release of lysosomal cathepsin L in microglia may contribute to inflammatory responses [142]. Upon pro-inflammatory stimulation, activated microglia also release cathepsin B, which has been shown to be a major causative factor of microglia-induced neuronal apoptosis [27]. Cathepsin B promotes neurotoxic polarization of microglia, for which two differential mechanisms have been suggested. One is the direct killing of neurons by cathepsin B secreted from neurotoxic microglia [27]. The other is that cathepsin B is involved in the production and secretion of inflammatory mediators from M1 polarized microglia [29]. The latter mechanism is more likely, as cathepsin B inhibition by CA-074 failed to block neuronal death [56]. Likewise, cathepsin C aggravates neuroinflammation by promoting microglia polarization towards the M1 phenotype [149].

Recent studies have shown that cathepsin X is also strongly associated with microglia polarization towards the neurotoxic phenotype. The proteolytic activity of cathepsin X in culture supernatants of activated microglial cells can be evoked by LPS stimulation. Upregulated expression and increased release and activity of microglial cathepsin X can lead to neurotoxicity mediated by microglia activation [29,42]. We demonstrated that the specific irreversible cathepsin X inhibitor AMS36 reduces excessive release of nitric oxide, a marker of activated microglia, whereas it does not affect the basal nitric oxide level. Furthermore, cathepsin X inhibition with AMS36 reduced the LPS-induced elevated IL-6 and TNF-α levels in BV2 cell culture supernatants [42], indicating cathepsin X as a potential therapeutic target for neuroinflammation-induced neurodegeneration.

Alternatively, microglia can assume an M2 phenotype evoked by IL-4 or IL-13 that is characterized by thin cell bodies and branched processes. This could improve phagocytotic function and release numerous protective and trophic factors, triggering anti-inflammatory and immunosuppressive responses [150]. IL-4-stimulated microglia generally produce less nitric oxide and more L-proline and type-2 cytokines (e.g., IL-10 and TGF-β) that help promote tissue repair and ECM reconstruction [151,152]. IL-4 increases cathepsin S expression in primary cultured rat microglia and is involved in microglial migration and invasion [85]. This indicates a regulatory role of cathepsin S in the migration of microglia to a site of inflammation via ECM degradation [84].

### 4.3. Cysteine Cathepsins Trigger Neuroinflammatory Signaling

A growing body of evidence shows that TLRs, which recognize a wide variety of danger signals and activate inflammatory cascades [153] and their downstream signaling molecules, modulate microglial responses during acute neuroinflammation [154]. The inflammatory stimulus LPS is a major component of the cell wall of Gram-negative bacteria and is recognized by a receptor complex that consists of TLR4/myeloid differentiation protein 2 and CD14 [155,156,157]. Conversely, poly(I:C) is a synthetic analog of double-stranded RNA that can be generated during the replication of RNA and DNA viruses (18) and is mainly recognized by the TLR3 receptor [158,159,160]. Furthermore, exposure to TLR4 agonist LPS leads to an increase in cathepsins B, L, K, S, H, and X [29,42,120]. As well as this, TLR3 and TLR4 co-activation results in increased inflammatory responses compared to individual TLR activation; poly(I:C) and LPS induce distinct patterns of pro-inflammatory factors together with different patterns of cathepsin X expression and activity. TLR co-activation decreases intracellular cathepsin X activity and increases cathepsin X localization at the plasma membrane together with extracellular cathepsin X protein levels and activity (Pišlar et al., under review). Additionally, cathepsins have been linked with regulating TLR3, which is processed by cathepsins within Loop1 of leucine-rich repeat 12. When proteolytic cleavage is inhibited by either a cathepsin inhibitor or Loop1 deletion, TLR3 can still be activated by poly(I:C) in many types of cell lines that express transiently transfected TLR3. Moreover, unprocessed TLR3 is degraded more rapidly than processed TLR3 fragments, suggesting that the cathepsin-mediated proteolytic processing of TLR3 increases TLR3 stability [161].

Pro-inflammatory cytokines, TLRs, and other stress-like stimuli activate NF-κB [162], a transcriptional factor that regulates the innate inflammatory response [56]. Proteolytic relay of cathepsin B along with lysosomal aspartic peptidase cathepsin E activates NF-κB in activated microglia. Cathepsin E increases cathepsin B expression in microglia after hypoxic-ischemic brain damage in neonatal mice through proteolytic modulation of TNF-related apoptosis-induced ligand (TRAIL), which in turn activates NF-κB in a proteasome-dependent manner. Conversely, in activated microglia following hypoxic-ischemic brain injury of neonatal mice, cathepsin B-mediated autophagy machinery promotes the degradation of NF-κB inhibitor alpha and subsequent NF-κB nuclear translocation [56]. Thus, the critical role of the proteolytic relay through the early cathepsin E/TRAIL-dependent proteasomal and late cathepsin B-dependent autophagic pathways for NF-κB activation has been suggested as a phenotypic switch in microglia along with the M1-M2 phenotypes [56,132]. Other cathepsins are also involved in the NF-κB pathway, namely cathepsins L and X. Cathepsin L inhibition alleviates microglia-mediated neuroinflammatory responses through the caspase-8 and NF-κB pathways [135]. Similarly, the impact of cathepsin X on the molecular pathways mediated by neurotoxin 6-hydroxydopamine is reflected in the NF-κB pathway. Neurotoxin-induced NF-κB nuclear translocation was decreased by the cathepsin X inhibitor AMS36, which coincided with the blocked degradation of NF-κB inhibitor alpha [121].

Cysteine cathepsins have also been linked to the mitogen-activated protein kinase (MAPK) signaling pathway in microglia. The inflammatory response elicited by activated microglia is associated with MAPK activation, and this can lead to a variety of physiological processes, such as cell growth, differentiation, and apoptotic cell death [163]. The MAPK family, which includes c-Jun N-terminal kinase (JNK), p38, and extracellular signal-regulated kinase (ERK), plays a critical role in the production of cytokines and mediators associated with the pathogenesis of inflammation [164]. Indeed, LPS induces p38, JNK, and ERK activation in BV2 cells [165,166]. Under LPS stimulation, cathepsin C enhances microglia activation and production of IL-1β and TNF-α, and this activation occurs through the phosphorylation of p38 MAPK, thus aggravating neuroinflammation. In this way, triggered activation of the Ca^2+^-dependent protein kinase C/p38 MAPK/NF-κB cascade controls a range of cellular processes, including chemotaxis, phagocytosis, and cytokine secretion [149]. Additionally, cathepsin X is a modulator of the MAPK signaling pathway in activated microglia. Inhibition of excessive cathepsin X proteolytic activity by AMS36 in LPS-activated BV2 cells markedly blocked LPS-induced p38 and JNK activation and reduced LPS-induced phosphorylation of ERK1 and ERK2, suppressing the increased cytokine release from activated microglia [42]. 

Furthermore, p38 MAPK is activated in damaged areas in many neuroinflammation-related diseases, including lysosomal storage diseases. In Niemann-Pick disease type C (NPC), which is characterized by intracellular accumulation and redistribution of cholesterol in several tissues, including the brain [167], cathepsins B and L are recognized as major lysosomal peptidases that control lysosomal function. Inhibition of cathepsins B and L leads to lysosomal impairment. Furthermore, loss of cathepsin B and L activity leads to the accumulation of free cholesterol in late endosomes/lysosomes, resembling a phenotype characteristic of NPC [168]. However, an in vitro study revealed that intracellular cholesterol accumulation induced by the NPC1 mutation enhances cysteine cathepsin S expression via abnormal p38 MAPK activation in microglia, and in turn stimulates Cx3cl1-Cx3cr1. In the CNS, Cx3cl1 (also known as fractalkine) is constitutively produced by neurons and binds to its receptor Cx3cr1 on microglia [169]. Cathepsin S-mediated Cx3cl1 secretion seems to be crucial for the development of neuropathic pain, because inhibition of cathepsin S activity facilitates pain control in a peripheral nerve injury model [26,170]. Seo et al. therefore addressed the significance of Cx3cl-Cx3cr1 interactions in the development of microglial neurotoxicity, in which cathepsin S has been suggested as a key upstream regulator [171]. Taken together, alterations in the expression and activity levels of microglial cysteine cathepsins affect inflammatory signaling, which is reflected in the severity of neuroinflammation.

## 5. Conclusions and Future Perspectives

It is widely accepted that neuroinflammation is an important factor in the pathogenesis of several neurodegenerative diseases and that the process is driven by activated microglia, which release pro-inflammatory mediators into the neuronal environment. Microglia-derived cysteine cathepsins are recognized as important pro-inflammatory mediators, triggering signaling pathways in inflammation-related cascades. Distinct cathepsins are upregulated in brain cells in the CNS during neurodegenerative pathologies associated with inflammation. Certain cysteine cathepsins have been found to be highly expressed and secreted from activated microglia, where a more detailed role of cathepsins in the microglia polarization towards M1 phenotype has been proposed. As cysteine cathepsins exhibit increased expression, activity, and subsequent secretion from activated microglia and participate in neuroinflammation-induced neurodegeneration, these peptidases are identified targets for the development of new diagnostic and therapeutic interventions in patients with neurodegenerative diseases. The beneficial effects of cystatins, endogenous cathepsin peptidase inhibitors, have yet to be demonstrated in neurodegenerative pathologies. Nevertheless, as they are general inhibitors, i.e., not cathepsin-specific, they can be expected to show off-target side effects. Therefore, rather than cystatins and other endogenous cysteine peptidase inhibitors, studies to date have focused on small synthetic inhibitors of appropriate specificities, with an emphasis on inhibiting excessive proteolytic activity of cysteine cathepsins associated with neuroinflammation-induced neurodegeneration. The design of selective and reversible cathepsin inhibitors is expected to improve peptidase-targeted therapy, which could significantly improve the treatment of patients with neurodegenerative disorders.
